# Experience, Prevalence, and Severity of Dental Caries in Mexican Preschool and School-Aged Children

**DOI:** 10.7759/cureus.51079

**Published:** 2023-12-25

**Authors:** Crystel G Vera-Virrueta, Fátima Sansores-Ambrosio, Juan F Casanova-Rosado, Mirna I Minaya-Sánchez, Alejandro J Casanova-Rosado, Juan A Casanova-Sarmiento, Saraí C Guadarrama-Reyes, Rubén de la Rosa-Santillana, Carlo E Medina-Solís, Gerardo Maupomé

**Affiliations:** 1 School of Dentistry, Autonomous University of Campeche, Campeche, MEX; 2 Faculty of Dentistry, Autonomous University of the State of Mexico, Toluca, MEX; 3 Academic Area of Dentistry of Health Sciences Institute, Autonomous University of Hidalgo State, Pachuca, MEX; 4 Advanced Studies and Research Center in Dentistry "Dr. Keisaburo Miyata" School of Dentistry, Autonomous University of the State of Mexico, Toluca, MEX; 5 Department of Epidemiology, Richard M. Fairbanks School of Public Health, Indiana University, Indianapolis, USA

**Keywords:** child, preschool child, permanent dentition, deciduous tooth, dental caries, oral health

## Abstract

Objective

Dental caries in Mexico continues to be a public health problem, indicated by it is high prevalence and incidence in children. This disease is associated with pain in preschool and school children, with large-scale consequences for the individual, society, and health systems. The objective of the present study was to determine the experience (mean of caries indices), prevalence, and severity of caries in children 2-12 years of age who sought dental care at a dental school in Mexico.

Material and methods

A cross-sectional study was carried out on 826 clinical records of patients ages 2-12 years. The dependent variable was caries, in terms of experience (mean primary teeth (dmft) and permanent teeth (DMFT) indices), prevalence (dmft and DMFT greater than 0), and severity (cutoffs of caries indices at various levels). The independent variables were age and sex. The data were analyzed in Stata 14 (StataCorp LLC, College Station, Texas).

Results

The average age was 7.2±2.3; 52.4% were boys. In the primary dentition, the caries experience (mean dmft) was 3.01±3.30, the prevalence of caries was 65.8%, and the severity dmft>3=37.3% and dmft>6=15.6%. In the permanent dentition, the caries experience (mean DMFT) was 0.99±1.88, the prevalence of caries was 31.5%, and the severity DMFT>3=12.5% ​​and DMFT>6=1.6%. The percentage of caries-free children in both dentitions was 26.1% (n=216/826). The experience, prevalence, and severity of caries were statistically different by age (p<0.001). Only in the severity of dmft>3 were differences observed across sex (p<0.05). Among children with mixed dentition, primary teeth were more affected than permanent teeth (2.46±2.87 vs 0.88±1.61; p<0.0001).

Conclusions

About seven out of 10 children were affected by cavities in either or both dentitions. It was observed that age was positively associated with dental caries, equally affecting girls and boys. In this sample, the primary dentition was impacted more than the permanent dentition. Despite being preventable, dental caries continues to be a health problem in children.

## Introduction

According to the World Health Organization's Global Oral Health Status Report (2022), oral diseases impact around 3.5 billion people worldwide, with three out of every four persons who are affected residing in middle-income countries. Preventive and clinical care are more problematic for approximately 514 million children who have caries in primary teeth, and two billion people worldwide have caries in permanent teeth; this is a serious public health problem [1,2]. Dental caries is also a common disease in Mexico, affecting people of all ages, but it mainly impacts those with greater social disadvantage, becoming an unresolved issue in health inequalities. The high prevalence and incidence of dental caries in Mexico not only have a significant impact on individuals and society in general in terms of suffering, pain, loss of daily activities, and productivity but also represent a significant financial burden for the healthcare system due to the high costs of treatment [3,4].

Mexico has one of the highest rates of dental caries in the world. According to data from two national studies [4,5], around 50% of students aged 5-16 have or reported having dental caries. Other estimates mention that between 70.0% and 85.0% of 12-year-old children have caries in permanent dentition, and 50.0% of 6-year-old children have caries in primary dentition [6]. In a recent systematic review in 2020, the worldwide prevalence of dental caries in primary teeth was estimated to be 46.2% (95% CI: 41.6-50.8%), and in permanent teeth was 53.8% (95% CI: 50.0-57.5%) [7]. In African countries, the prevalence of dental caries among 12-year-old individuals is 36.0% (95% CI: 29.4-41.7%) [8]. In Latin America, the prevalence of dental caries in primary dentition is 56% (95% CI: 52-59%), and in permanent dentition is 58% (95% CI: 54-61%) [9].

Dental caries is a condition caused by a disturbance in the balance of microorganisms in the mouth, which is influenced by various factors and primarily driven by sugar consumption. This leads to a demineralization and remineralization process of the hard tissues of the teeth, as well as their eventual dissolution due to the acid produced by specific bacteria in the mouth. The interplay between pathogenic and protective variables determines the onset and advancement of dental caries. Dental caries is a preventable disease that is not spread uniformly and has significant economic and quality of life impacts [10,11]. Dental caries is a ‘deprivation disease’ that follows a socioeconomic gradient, whereby people at a social disadvantage are affected the most - including higher caries prevalence, worse caries rates, and unmet caries care needs [12]. Latin America is a region of stark contrasts, where wealth and prosperity coexist with vulnerability and extreme poverty. Mexico is one of the countries in Latin America with higher inequality in the region, as only 10% of the population concentrates 59% of the country's income and 1% accounts for 29% [13]. These economic inequalities are associated with health [14], where people with lower socioeconomic status have the worst oral health conditions but the least access to healthcare services. In addition, dental caries in primary dentition affects the well-being of young children and is an important predictor of caries in permanent dentition. Unfavorable scenarios in children's oral health lead to adverse situations in adolescents and adults in the future [15-17].

The objective of the present study was to quantify the experience (mean of caries indices), prevalence, and severity of caries in children 2-12 years of age who sought care at a dental school in Mexico. The hypotheses employed were the assumptions that (separately) experience, prevalence, and severity of caries would be generally at the poorer end of their ranges.

## Materials and methods

Study design, population, and sample

A cross-sectional, observational, retrospective study was conducted on clinical records of patients aged 2-12 years who attended the dental clinics in the School of Dentistry at the Autonomous University of Campeche. (All public universities in Mexico are considered autonomous by default.) The sample size was calculated with a 95% confidence level, 3% precision, and a proportion (approximate parameter value) of 75% [18], resulting in 800 individuals. An expected dropout proportion of 5% was added (842 individuals). Clinical records are compiled by students who have been standardized under the supervision of faculty members.

Inclusion and exclusion criteria

The inclusion criteria were 1) clinical records of individuals of either sex, 2) aged 2-12 years, 3) attending the pediatric dentistry service, 4) clinical records approved by the clinical faculty, and 5) signed informed consent. The exclusion criteria were 1) incomplete clinical records, 2) missing data in the clinical records, 3) lacking clinical records at the time of the study, and 4) canceled clinical records. After applying the inclusion and exclusion criteria, the final sample size for the analysis in the present study was 826.

Included variables

The dependent variables were the experience, prevalence, and severity of dental caries for both dentitions. Caries experience is based on indices that quantify the current (caries component) and past (missing and filled components) experiences in their components [19]. Dental caries presence was diagnosed through the common clinical examination conducted for filling out the clinical records. The indices were obtained from the diagnostic records in the clinical records. The experience of caries in the primary dentition refers to the group average of decayed (d), missing (m), and filled (f) teeth in the primary dentition, known as the dmft index and obtained using the formula:

dmft=∑ dt+ mt+ft of all individuals / total number examined

In permanent dentition, the experience of caries refers to the group average of decayed (D), missing (M), and filled (F) teeth in the DMFT index, obtained using the formula:

DMFT=∑ DT+ MT+FT of all individuals / total number examined

Caries prevalence for each dentition was coded as 0 = if dmft or DMFT was equal to 0 and 1 = if the dmft or DMFT > 0, for each dentition. Similarly, to calculate caries severity, two cutoff points were established:

1) Low caries severity: for both dentitions, coded as 0 = if in the dmft or DMFT ≤ 3 and 1 = if the dmft or DMFT > 3, for each dentition, and

2) High caries severity: for both dentitions, coded as 0 = if in the dmft or DMFT ≤ 6 and 1 = if the dmft or DMFT > 6, for each dentition.

The independent variables were age and sex.

Statistical analysis

For quantitative variables, measures of central tendency and dispersion were calculated, and for categorical variables, frequencies and percentages were determined. Non-parametric tests, such as the Mann-Whitney U test, Spearman's correlation, and the chi-square test, were used for bivariate analysis. All analyses were performed using Stata 14.0® software (StataCorp LLC, College Station, Texas).

Ethical considerations

The study protocol was approved by the Ethics and Research Committee of the Faculty of Dentistry at the Autonomous University of Campeche (IRB 09-2021-4). This research was conducted following the applicable General Health Law in Mexico.

## Results

The results of the sample distribution by age and sex are shown in Table 1. The final sample of children included in the study was 826. The average age was 7.2±2.3, with 52.4% being boys. Of the total individuals included, 96.7% (n=799) had primary dentition, 75.2% (n=621) had permanent teeth, and 594 (71.9%) had mixed dentition. Of note is that 26.1% (n=216/826) (95% CI: 23.1-29.1) were caries-free in both dentitions.

**Table 1:** Distribution of the sample by age and sex SD = standard deviation

	Mean	SD
Age (years)	7.2	2.3
	Frequency	Percentage
Sex		
Girls	393	47.6
Boys	433	52.4

Caries in primary dentition

Table 2 shows the results of caries experience (mean dmft index), prevalence, and severity in primary dentition. The mean dmft index was 3.01±3.30 (95% CI: 2.78-3.24), the prevalence of caries (dmft>0) was 65.8% (95% CI: 62.5-69.1), and severity, for dmft>3 was 37.3% (95% CI: 33.9-40.6), and dmft>6 was 15.6% (95% CI: 13.1-18.1). At six years of age, the dmft index (caries experience) was 3.43±3.46 (95% CI: 2.87-3.99), and the prevalence of caries was 68.6% (95% CI: 61.1-76.1). The "decayed teeth" component constituted the largest proportion of the dmft index, at 88.0%.

**Table 2:** Distribution of the dmft, DMFT indices (caries experience), and their components dmft = decay, missing and filled teeth in primary dentition (caries experience in primary dentition) DMFT = Decay, missing, and filled teeth in permanent dentition (caries experience in permanent dentition)

dmft (n=799) (mean±sd)	decay (mean±sd)	missing (mean±sd)	filled (mean±sd)	% dmft > 0	% dmft > 3	% dmft > 6
3.01±3.30	2.65±3.12	0.32±0.84	0.03±0.29	65.8	37.3	15.6
DMFT (n=621) (mean±sd)	Decay (mean±sd)	Missing (mean±sd)	Filled (mean±sd)	% DMFT > 0	% DMFT > 3	% DMFT > 6
0.99±1.88	0.95±1.78	0.01±0.14	0.02±0.34	31.5	12.5	1.6

Caries in permanent dentition

Similarly, Table 2 shows the results of caries experience (mean dmft index), prevalence, and severity in permanent dentition. The mean DMFT index (caries experience) was 0.99±1.88 (95% CI: 0.84-1.14), the prevalence of caries (DMFT>0) was 31.5% (95% CI: 27.8-35.2), and severity (DMFT>3) was 12.5% (95% CI: 9.9-15.1), and DMFT>6 was 1.6% (95% CI: 0.6-2.6). At 12 years of age, the DMFT index was 3.13±4.16 (95% CI: 1.57-4.68), and the prevalence of caries was 66.6% (95% CI: 48.7-84.5). The "decayed teeth" component constituted the largest proportion of the DMFT index, at 95.9%.

Results of the distribution by age and sex

The results of caries experience (mean dmft/DMFT indices) in both dentitions are shown in Table 3. In the Spearman correlation test, it was observed that, as age increased, the dmft decreased (r=-0.3679, p<0.0001), and DMFT increased (r=0.3690, p<0.0001). No differences in caries experience in either dentition by sex were observed (p>0.05).

**Table 3:** Distribution of caries experience by age and sex dmft = decay, missing and filled teeth in primary dentition DMFT = Decay, missing, and filled teeth in permanent dentition *Spearman correlation †Mann-Whitney test Significant p-value < 0.05

	dmft index (mean±sd)	p-value
Age (years)	r = -0.3672	<0.0001*
Sex		
Girls	2.79±3.12	z = -1.134
Boys	3.20±3.46	p= 0.2568†
	DMFT index (mean±sd)	p-value
Age (years)	r = 0.3657	< 0.0001*
Sex		
Girls	1.08±2.10	z = 0.525
Boys	0.91±1.65	p= 0.5997†

Table 4 shows the results regarding the prevalence of caries in primary and permanent dentition. In the Mann-Whitney test, it was observed that the average age was lower in children who had caries in primary dentition compared to those who did not have caries (age 6.56±1.97 with caries vs 7.97±2.37 without caries; p<0.0001). On the other hand, the average age was higher in children who had caries in permanent dentition compared to those who did not have caries (age 9.09±1.79 with caries vs 7.73±1.70 without caries; p<0.0001). No differences in the prevalence of caries in primary and permanent dentition by sex were observed (p>0.05).

**Table 4:** Distribution of caries prevalence by age and sex *Mann-Whitney †Chi-square Significant p-value < 0.05

Primary dentition	Without caries	With caries	p-value
Age (years) (mean±sd)	7.97±2.37	6.56±1.97	z = 8.287
			p <0.0001 *
Sex	n (%)	n (%)	
Girls	126 (33.2)	253 (66.8)	
Boys	147 (35.0)	273 (65.0)	0.602 †
Permanent dentition	Without caries	With caries	p-value
Age (years) (mean±sd)	7.73±1.70	9.09±1.79	z = -8.615
			p < 0.0001
Sex	n (%)	n (%)	
Girls	203 (67.6)	97 (32.3)	
Boys	222 (69.1)	99 (30.8)	0.689†

The results of caries severity in primary and permanent dentition can be seen in Table 5. Using the Mann-Whitney test, it was observed that the age in children with caries severity in primary dentition (dmft>3 and dmft>6) was lower than their counterparts (p<0.0001), while the average age was higher in children with higher caries severity in permanent dentition (DMFT>3 and DMFT>6) (p<0.0001). Regarding the analysis by sex, differences were only observed in the severity of dmft>3 (p<0.05).

**Table 5:** Distribution of caries severity by age and sex dmft = decay, missing and filled teeth in primary dentition DMFT = Decay, missing and filled teeth in permanent dentition *Mann-Whitney †Chi-square Significant p-value < 0.05

Primary dentition	dmft ≤ 3	dmft > 3	dmft ≤ 6	dmft > 6
Age (years) (mean±sd)	7.57±2.26	6.15±1.82	7.31±2.21	5.60±1.57
	z = 8.866, p < 0.0001*		z = 8.001, p < 0.0001*	
Sex	n (%)	n (%)	n (%)	n (%)
Girls	254 (67.0)	125 (33.0)	327 (86.3)	52 (13.7)
Boys	247 (58.8)	173 (41.2)	347 (82.6)	73 (17.4)
	p = 0.017†		p = 0.155†	
Permanent dentition	DMFT ≤ 3	DMFT > 3	DMFT > 6	DMFT > 6
Age (years) (mean±sd)	7.95±1.77	9.60±1.64	8.12± 1.82	10.50± 1.64
	z = -7.215, p < 0.0001*		z = -3.602, p = 0.0003	
Sex	n (%)	n (%)	n (%)	n (%)
Girls	258 (86.0)	42 (14.0)	294 (98.0)	6 (2.0)
Boys	285 (88.8)	36 (11.2)	317 (98.8)	4 (1.2)
	p = 0.295		p = 0.456	

In children with mixed dentition, primary teeth were more affected than permanent teeth (2.46±2.87 vs 0.88±1.61; p<0.0001).

## Discussion

The study demonstrated the presence of high caries experience and high percentages of prevalence and severity, mainly in the primary dentition. Since the percentage of caries indices in both dentitions is mainly composed of the "decayed" component, it demonstrates high treatment needs for caries. Similar situations have been found in Latin America [20-22], in Mexico [3,4,23-25], and in other countries around the world with similar populations [8,9,26,27]. In children living in middle-income countries, such as Mexico, some factors contribute to the presence and development of dental caries, including lack of proper dental hygiene, a diet rich in cariogenic sugars, lack of access to oral health services, and additional preventive measures, such as fluoride supplements. All of these result in a large amount of caries in both primary and permanent dentition [28,29].

Caries in primary dentition

The present study revealed that the prevalence of caries in the primary dentition and the mean dmft among children aged 2-12 years (65.8%, 3.01), specifically at six years of age (68.6%, 3.43), were much higher than the national average in Mexico for six-year-old children (59.4%, 2.52) [8]. However, it is necessary to highlight certain nuances, as variations can be observed among the different states that make up the country, with caries prevalence figures in children aged six to eight years ranging from 36.4% to over 80% [4]. Another report [18], with a clinical sample, at the national level in Mexico, from 2005 to 2014, indicated that the caries index (dmft) in primary dentition ranged from 3.8 to 5.1 in children aged two to four years and from 3.8 to 4.7 in those aged five to nine years, with prevalences ranging from 66.2% to 82.1% and from 71.7% to 79.5% in each age group, respectively. Additionally, the prevalence of caries in the present sample is higher than in other recent surveys in Mexico [24]. Dental caries in the primary dentition was also lower when compared to what has been reported by other authors with clinical samples similar to ours, for example, in Hernández-Martinez et al., [23] in the State of Mexico, where an average dmft index of 8.53 and a caries prevalence of 99% were observed in children aged 2-12 years, or in Juárez-Zapata et al. [25] in Hidalgo who reported an average caries index of 5.19 and a prevalence of 77.7% in children aged 1-12 years.

A systematic review in Latin America estimated that dental caries in the primary dentition is 56% (95% CI: 52-59%), a figure lower than that observed in the present study, but with prevalences ranging from 36% to 71% only in Brazil or from 41% to 87% in other Latin American countries [9].

Caries in permanent dentition

The results of the present study revealed that the prevalence of caries in the permanent dentition and the mean DMFT index among 12-year-old children (66.6%, 3.13) were higher than the national average in Mexico for 12-year-old children (58.0%, 1.91) [4], although lower for the total sample of 5-12 years in the present study (31.5%, 0.99). However, this may be associated with certain variability in caries prevalences and DMFT indices by state, with DMFT figures ranging from 0.52 to over 3.67 [4]. According to the Department of Health [26], in individuals seeking health services at the national level from 2005 to 2014, the DMFT index ranged from 2.9 to 4.5 in adolescents aged 10-14 years, with prevalences ranging from 61.6% to 78.0%. Those figures are like those observed in the present study.

The prevalence of caries in the permanent dentition of this sample is higher than the results of recent surveys in Mexico [24], as well as in samples of individuals self-referred to clinical services. For example, Hernández-Martinez et al., [23] in the State of Mexico, reported an average DMFT index of 1.91 and a caries prevalence of 58.9% in children aged 2-12 years. Juárez-Zapata et al., [25] in Hidalgo, reported an average caries index of 1.18 and a prevalence of 35.1% in children aged 1-12 years.

Quoting again the systematic review in Latin America, dental caries in the permanent dentition in adolescents aged 11-13 years was 58.0% (95% CI: 54-61%), a figure equal to that observed in the present study, but with prevalences ranging from 27% to 90% in Brazil or from 23% to 93% in other countries in Latin America [9]. For the sake of adding a simple geographic contrast, in African countries, the prevalence of caries in 12 year olds is 36.0% (95% CI: 29.4-41.7%) [8], a figure much lower than that reported in the present study.

Components of dental caries indices

The proportion of each component of the dmft index in primary dentition, and DMFT in permanent dentition, has implications for the affected child and is an indicator of oral health care for the population in question [27]. Although some studies report high prevalences of restorative experience for dental caries [30], most reports relevant to Latin America [9] and other middle-income countries [15,26] showed a higher proportion of the "decayed" component in both primary and permanent teeth. Finding children suffering from untreated caries-induced pain is a common occurrence in these countries, such as Mexico [31]. This has an impact on the quality of life of children, affecting those with lower socioeconomic status more: large numbers of children would benefit from better access to dental prevention and care.

The observed differences in caries indices in primary and permanent dentition between different reports and our findings may be due to different situations, for example, the age range of the individuals studied, the type of populations included, and even the development of the community or the country where the study was conducted. Dental caries can be effectively prevented and controlled through simple and cost-effective actions, such as tooth brushing, the placement of pit and fissure sealants, and the use of technologies such as public health fluoridation, thereby preventing it from progressing to cavitated lesions while preserving dental structure [32-36]. However, the effectiveness of preventive measures can be affected by the strong correlation between caries and socioeconomic factors. It is worrisome that inequalities in oral health prevail in Mexico [3,4,23-25,37-39]. Prevention measures to ameliorate the onset and progression of caries should be implemented. In addition to tooth pain and sensitivity, premature tooth loss, and chronic dental infections caused by this disease can stress the immune system and have a negative impact on a person's overall well-being. At very advanced stages, caries can even lead to death [40,41].

There are some strengths and limitations of the study, hereby described to better situate its messages. The study adds to the body of evidence connoting high disease experiences and considerable unmet treatment needs in Mexican children. Contrasts across age groups and biological sex help characterize the burden of disease in sub-groups. The information obtained can be applied to help design preventive and health promotion programs, as well as fine-tune prevailing trends in caries experience. One limitation of the study is the type of sample included, as they were seeking dental care; while comparisons between our results show some similarity with other studies, the sample in the present study does not represent the general population. Another limitation is that the study is a retrospective, secondary analysis of existing clinical records; students were trained to communicate with patients and enter data, but no strict calibration of students nor Kappa-based assessments were undertaken.

## Conclusions

In conclusion, about seven out of 10 children in our self-selected sample were affected by caries in either primary or permanent dentition. High unmet treatment needs for caries were observed in both dentitions: older age was associated with dental caries, affecting girls and boys about equally. The primary dentition was more severely affected than the permanent dentition.

To start addressing such oral health and dental care patterns, it appears necessary to implement public health strategies (local and national) actively supporting good oral health outcomes in preschool and school-age children. These community strategies could include placing pit and fissure sealants or fluoride varnish in schools, which have proven to be a cost-effective strategy for reducing dental caries. At a larger scale, the results generally support a call to increase the overall level of awareness about the importance of oral health and the major influences on oral health (such as industrial determinants of health embodied in inordinate snack consumption, the roles of sugars in dental caries, and the ubiquitous use of sugared beverages). Together with pointers leading to early identification of clinical problems, age-specific and relevant educational materials could help strengthen self-care practices, as well as (when feasible) opting for lifestyle choices that are less aggressive to teeth. Improved access to timely, affordable, and sufficient clinical care for the population at large ought to complement larger health policy perspectives and priorities.
